# The Case of the Abbe Mann

**Published:** 1804-03-01

**Authors:** 


### The Case of the Abbe Mann.

THE following case of severe Gout, successfully treated, has excited much attention on the Continent; and from the respectability of the channel through which it has been received, we cannot doubt the fidelity or accuracy of the relation. Our readers will perceive the language of the humoral pathology, which still prevails on the Continent, and some theoretical opinions which are obsolete here; but we did not think ourselves justified in any alteration.

Some have doubted whether this was a case of simple genuine gout; our readers will exercise their own judgments on this subject, and no doubt all will agree that the sufferings of the patient were uncommonly severe, and the success of the remedies was so complete, as to entitle them to farther trials in similar affections.

---

To

*To the Editors of the Esprit des Journaux.*

Gentlemen, *Brussels, Dec.* 31, 1803.

The very extraordinary change in the state of my health, which I have experienced within a few years, has induced many of my friends, who have witnessed it, earnestly to request me to prepare the following statement of the means by which this improvement has been produced. Having been advised by them, and by several medical practitioners, to communicate it to the public, in the hope that others, suffering under the complaint with which I was so long afflicted, may derive some benefit from the communication, I must beg, if you should entertain the same opinion, that it may be inserted in your Journal.

I have the honour, &c. &c.

The Abbe Mann, Canon of Courtray, &c.

*Account of a remarkable Case of inveterate Gout, cured by a long Exhibition of Extract of Hemlock, and of Aconite or Monkshood, prepared according to the Prescription of M. Storck.*

The Abbé Mann had been accustomed, in his youth, to a life of great personal activity. At a very early age, he had acquired a decided taste for study, and after passing through the usual course of education, continued to devote, to scientific pursuits, every interval of leisure of which he could avail himself amidst the duties of his profession, and during long and frequent marches. This predilection for study, and the love of solitude which it naturally inspired, determined him, at the age of twenty-five years, to leave Spain, and to quit the army, with a view to enter into a Carthusian Order in Flanders.

In 1763, in the twenty-ninth year of his age, he was affected with arthritic pains, which, at times, were somewhat acute, and continued for several months, without his being able distinctly to ascertain their cause. The pains in the feet were not so considerable as to prevent him from walking; but he was scarcely ever entirely free from them, either during the winter of 1763 and 1764, or in the following spring. About this time he was nominated Prior to the Carthusian Monastery at Nieuport. A few days afterwards, he became seriously indisposed with a continued fever, which was shortly succeeded by a regular fit of the gout in both feet. The fever continuing with some degree of violence, his physician improperly directed him to be bled no less than three times. This mode of treatment

 produced produced an effect, which might easily have been foreseen. The gout was removed from the feet; but the patient experienced nausea, faintness, and sickness, of which he never felt any thing while the gout was confined to the extremities. This indisposition continued six weeks, and the Abbé recovered his health with great difficulty, and very slowly.

During the remainder of the year 1764, and in the spring of 1765, he had several slight fits of the gout. They sometimes affected the feet, but, for the most part, were fluctuating and irregular.

The duties of his profession, and his private affairs, rendering it necessary for him, after his recovery, to take continual exercise, either on horseback or on foot; his constitution became so considerably strengthened, that, during a space of two years and a half, he had no return of the gout, and experienced no serious illness of any description.

Before entering on any detailed account of the remedy to which the Abbé had recourse, it may not be improper to point out, in a few words, the most apparent predisposing causes of his very arthritic constitution. According to *his best judgment*, they appeared to him to be the following.

1. From the year 1756 to 1764, he studied with intense application, seldom less than thirteen or fourteen hours daily; returning to this arduous employment immediately after his repasts, and frequently trespassing on the time which Nature has destined for repose.

2. After entering into the Carthusian Order, he had few opportunities of taking exercise.

3. The difference between the climate of Spain and that of the Flemish coast, and the extreme cold to which he was exposed in the winter season; being daily employed at church four or five hours, and often more, beside three hours at least during the night. Circumstances of this description could not fail of materially injuring the constitution.

4. The privation of that repose which Nature requires, especially after his appointment in 1764 to Superior of the Order. His rest was frequently limited to three or four hours, after one o'clock in the morning. In 1769, the very feeble state of his health compelled him, however, to change this practice.

5. Indefatigable application to study, prosecuted with as much and often more ardour at night, than during the day.

In the opinion of the Abbé Mann, these are the principal circumstances which rendered his constitution so susceptibly ceptibly arthritic, that, from the autumn of 1768 to the summer of 1779, he annually experienced two, three, or four attacks of this excruciating complaint. He may venture to assert, with the greatest truth, that, during this long interval, he has seldom enjoyed for more than three or four months in the year, a moderate degree of health, unaccompanied with pain, arising from regular or irregular symptoms of this disorder. A short account of some of the most severe attacks, with which he has been afflicted, will more distinctly explain the circumstances of his case.

In the month of September, 1768, he was attacked with the gout in the stomach, which continued a fortnight, with the ordinary symptoms of nausea, sickness, &c. &c. The disorder afterwards affected his chest and lungs, and occasioned much difficulty in respiration. His physician ordered that he should be twice bled in the feet; but this, for some time, only aggravated the complaint.

In January, 1770, he was obliged to proceed, on private affairs, to Brussels and Molines. The season was extremely severe, and he felt the symptoms of an approaching fit of the gout. The pain which he endured during the journey, was inexpressibly acute; especially in the stomach and the kidnies. Upon this occasion, he was afflicted with the gravel, which afterwards accompanied every attack of this complaint. It was with great difficulty, and inconceivable pain, that he returned to Nieuport, after an absence of three weeks.

His physician directed cataplasms to be applied, which fixed the gout in his feet and right hand, where it continued, with the regular symptoms, four or five weeks. The cataplasm, applied to the left ancle, drew the arthritic humours so powerfully to that part, that they pierced the skin above the inner bandage, and continued in a state of discharge for several months, producing in the left leg a degree of weakness, of which the Abbé is still sensible. In March, and at a time that he thought the fit had left him, the gout returned; attacking the viscera, the stomach and the kidnies. This paroxysm returned every eight or ten days, till April 22, when it was accompanied with the most violent and acute pains, excruciating nervous affections, and continual vomiting after taking medicine, food, &c. In this alarming state, in which his life was almost despaired of, he remained during four days and three nights, without an instant of repose. It is utterly impossible to convey an adequate idea of the agony which he endured. He had several relapses before the end of May, and and the frequent vomiting continued more than a twelvemonth afterwards. During the nine years the Abbé was afflicted with the gout, he was constantly subject, as will appear in the sequel, to indigestion and the most violent heart-burn.

In the two following years, he had several fits of the gout, for the most part regular, which affected the feet and hands, but were not accompanied with excessive pain. The right hand, and the right and left foot, were always first, and successively, attacked; and lastly, though seldom, the left hand. These paroxysms generally continued three or four weeks. But, at the beginning of 1772, he had a fit which lasted twice as long; affecting, at the same time, both feet and the right hand, and producing the most acute pain. This was one of the most violent regular fits of the gout which the Abbé ever experienced.

At the commencement of the summer of 1772, finding himself constantly in a state of fluctuating debility, and subject to continual indigestion, heart-burn, and violent sickness, he was advised by several of his friends, to have recourse to the regimen prescribed in Dr. Cadogan's treatise on the gout; a work which had acquired in London some degree of reputation. This regimen consists chiefly in taking exercise in the open air, avoiding every species of fermented liquor, and food that has a tendency in digestion to produce acidity. The Abbé was persuaded to try this mode of cure; and, notwithstanding the feeble and relaxed state of his stomach, continued the practice of drinking water for four months, and observed all the injunctions of the regimen, as far as the circumstances of his health would permit. During this interval, he twice felt the symptoms of an approaching fit, which even continued for a few days, and afterwards entirely disappeared, without producing any regular attack of this disorder. In the mean time he was reduced to a state of great debility, in which he continued till the end of September, when he was attacked with the gout in the viscera, accompanied with the most alarming symptoms. This dreadful fit, the most violent and dangerous which he ever experienced, continued through the winter till April 1773; a period of about seven months. It should be observed, that in 1770, the gout attacked the Abbé in the stomach and the abdomen. This fit was of a different description, for it affected the stomach, the chest, and the head.

The symptoms, which frequently recurred, of this latter species of arthritic attack, were, nausea, sickness at the stomach, stomach, and faintness. After remaining some hours in this state, the respiration became gradually impeded to so great a degree, as, at times, entirely to cease; renewing itself at intervals. He afterwards felt a suffocation and strangling, as if the neck was closely compressed with a bandage. This was succeeded by a sensation of a cold humour propelled towards the head, which successively affected the eyes, the ears, and the tongue. The latter swelled so considerably, that it entirely filled the mouth, and lost its muscular motion. Thus deprived of sight and of speech, he could only hear those who were near him, as if they were speaking at a distance. He entirely lost the power of motion, except in the fingers of the left hand, and almost ceased to have any external sensation. *His presence of mind, however, was not in the least affected.* In these circumstances, the strongest cataplasms, and afterwards, blisters, were applied to the feet. In order to recover him from this lethargic state, which, it was supposed, might produce either apoplexy or arthritic paralysis, the physicians ordered him to be shaken with some degree of violence, and the soles of his feet to be rubbed, although excoriated with the cataplasms and blisters. So long as he continued in this state of lethargy, or rather of privation of almost all external feeling, he was scarcely sensible of any impression from the violence of these operations. But, in proportion as he revived, these parts became affected with inexpressibly acute and shooting pains. In the space of about two months, he experienced above twenty attacks of this description; the shortest of which continued about eight hours, and the longest about eighteen. Upon more than one occasion, the physicians conceived him to be on the point of dissolution, and even expressed this opinion. The 1st of September it was supposed that he could not survive the night. A few doses of diaphoretic antimony (antimonium calcinatum) occasioned a sort of crisis, and produced a copious perspiration, which continued an hour, and probably saved his life, by thus promoting a discharge of arthritic humours. He was however, reduced to a state of extreme weakness. In the intervals of these internal attacks, the gout violently affected the feet, the legs as far as the knees, and the right hand and arm. The acute pains and nervous affections, which he endured, exceed all description. Nor did he suffer much less from arthritic colic and the gravel; and the humours which he discharged by vomiting were scarcely less acid than nitrous spirit.

In

In this afflicting situation, the Abbé remained during a great part of the time from September to December 1772. For three months afterwards, he had occasional fits of the gout; but they were considerably less violent, and did not affect the intestines. After this, he recovered his health gradually, but very slowly. During the space of two years, however, he felt, on the right side, from head to foot, a species of oppression and numbness, which evidently arose from this part having been frequently attacked with so many violent fits of this disorder; but, in time, these symptoms also disappeared.

In order to prevent the return of the gout, he was advised to have recourse to issues; and, in the autumn of 1773, he had an issue opened under each knee. Either from the effect which they produced, or from some other circumstance, the fits which he afterwards experienced became much less violent and dangerous, than those with which he had been attacked in 1770 and 1772. From this period to the year 1777, he had annually three or four fits of the gout, in the feet and the hands; but they were always more or less irregular, and accompanied with sickness at the stomach, nausea, and other symptoms, which indicated an arthritic affection of the intestines. These attacks generally continued three or four weeks, and were regularly succeeded by the gravel, which lasted a month or six weeks, attended with most acute pain, and a considerable discharge of red sand in the urinary secretions. When free from the gout and the gravel, he enjoyed but a moderate degree of health during three or four months of the spring and the summer. The remainder of the year, he suffered under a languishing and fluctuating debility, which terminated in the painful symptoms of an irregular arthritic affection, that seemed to attack the whole frame.

In the summer of 1777, the Abbé changed both his religious order and his residence. The late Empress-Queen had solicited, and obtained, from the Pope, the secularization of the Carthusian Order, which she afterwards established at Brussels. It might be supposed, that a change of climate, and a total change in diet, and in the mode of living, would have produced a material improvement in the state of his health. On the contrary, during the two following years, he was as much as ever afflicted with the gout, and its consequences. He was always either infirm, or attacked as formerly, with regular fits of this complaint.—Indigestion, heart-burn, and incapability of walking, or taking exercise, increased instead of diminishing.—

Half

Half an hour's exercise fatigued and exhausted him so much, as to endanger bringing on fainting fits. Every one advised him to take exercise, as the only remedy for his disorder; but he was entirely incapable of attempting it, from the stiffness of his joints, and his extreme weakness.

In this state he remained till the autumn of 1778, when he felt the predisposing symptoms of a violent arthritic attack. He was affected with the gout in the beginning of November. It extended to the viscera, the stomach, and the head, with symptoms and effects similar to those already described as accompanying the fit in October 1772. He continued some days in this state; the fit becoming afterwards more severe, it was judged proper to apply blisters to the calf of the legs. They had the good effect of drawing the arthritic humours from the intestines, and produced a partial discharge. But the Abbé remained long extremely faint, and in a very weak condition; and repeatedly experienced slight fits, after a few days interval, till the following spring. The capability of walking, or of taking any exercise, became more than ever enfeebled, and his powers of digestion so very weak, that all his food was converted into acrimony, and produced continual vomiting, sometimes even blood. His constitution had long been extremely relaxed; but this relaxation, since the last fit, was more considerably increased than ever. The diet he was obliged to observe, while in a state of convalescence, and during Lent in 1779, contributed without doubt to produce this effect. It may be farther remarked, that this excessive relaxation occasioned also a nervous affection, which he had never before experienced; at least, not, by any means, in so violent a degree. The whole nervous system seemed to be attacked by the acrid humours of the gout, producing spasms, nervous affections, and dreadful irritation. If the Abbé experienced more acute pain, in the severe fits with which he was afflicted in 1770 and 1772, than in the present, yet, what he then endured will admit of no comparison with the agony occasioned by this nervous irritation; for it affected the mind as much as the body, and rendered life itself insupportable.

Such was the condition to which the Abbé Mann was reduced in the spring of 1779; disgusted with physic and physicians, and entertaining but little hope, and even little desire, of a prolongation of life; which, in fact, was become a state of perpetual suffering. His situation interested in his favour a number of very respectable persons, who who induced M. Himmelbaur, a surgeon on the Staff of the Imperial Army, to visit him in the character of a Friend and Physician, and gradually to acquire his confidence. In this object, he succeeded towards the end of spring, and persuaded him to a total change of diet; prescribing, at the same time, medicines of a mild and sedative quality. After some time, the Abbé proposed to this skilful practitioner, the internal exhibition of hemlock, which he considered merely as a sedative. M. Himmelbaur approved of the proposition, and declared it to have been his wish to try this remedy, but that he had felt some difficulty in proposing it. He added, that, in his opinion, extract of hemlock was not only a sedative, but a powerful corrector of acridity of the humours, and well adapted to strengthen the powers of digestion. In consequence of this resolution, the Abbé began, in April, 1779, to take the extract of hemlock, prepared at Vienna in the manner prescribed by Baron Storck; to which M. Himmelbaur, some time after, added a just proportion of extract of aconite, or wolfsbane, prepared also at Vienna, according to the formula of the same celebrated Physician. From this place, the Abbé always procured these medicinal extracts. They were exhibited in the form of pills, containing each two grains.

He began by taking, three times a day, four pills of extract of hemlock. In proportion as he became accustomed to them, he increased the dose, taking at the same time a pill of extract of aconite. In five or six months, he had gradually augmented the daily dose to 100 and 120 grains; observing, however, the proportion of five or six pills of hemlock to one of aconite.

In these quantities he continues to take them, but in much smaller doses than during the two first years, when he was constantly exposed to arthritic affections. He afterwards added to the pills the following mixture prescribed by M. Himmelbaur.* Take of camphor, mucilage of gum Arabic, and sugar, each one drachm. Let them be well mixed, and add half a drachm of purified nitre, half an ounce of white wine vinegar, and six ounces of syrup of red poppies. He also took occasionally a dose of rhubarb. These are the only medicines which the Abbé has made use of during the last four years, confining, how-

---

* ℞. Camphoræ, mucilag. g. Arabici et sacchari, singulorum ʒj. bene tritis et mixtis, adde nitr. potassæ ʒß. acid. acetos. ℥ß. syrup. papav. errat. ℥vj. M. fiat mixtura.

ever, his regimen to nutritious food of easy digestion.—The following are the effects which resulted from this mode of treatment.

During the first three months, the hemlock produced no perceptible effect; he began even to despair of deriving any benefit from it. But M. Himmelbaur incessantly exhorted him to continue taking it, with an addition of extract of aconite. He followed this advice, and in a short time the stiffness of his joints was materially removed, and his capability of taking exercise considerably increased. The spasms and nervous affections began sensibly to diminish. Encouraged by this success, he zealously prosecuted this mode of treatment, and before the winter, he exhibited these extracts in the largest doses. His digestion became stronger, and the acrimonious humours in the stomach, with which he had been so many years afflicted, proportionately decreased. Great progress towards a cure was thus effected. During the winter of 1779 and 1780, the Abbé frequently suspected an approaching fit of this disorder. The winter, however, elapsed without any return of the gout, and, in the ensuing spring, he was able to walk with ease, his joints having acquired strength, and their stiffness being gradually removed. He availed himself of this improvement in his health to take more exercise, which he continued, without interruption, both in winter and in summer. But the most remarkable circumstance attending this mode of treatment relates to the powers of digestion. He now suffered but little from heart-burn and indigestion, though formerly he was perpetually subject to them. If, accidentally, he experienced any inconvenience from acrimonious attacks, a few pills of hemlock or of aconite immediately removed them, and in a more effectual manner than magnesia, or any other absorbent, without any of the consequences which magnesia usually produced.

During the winter of 1780 and 1781, he felt several symptoms of the gout, but without experiencing any fit whatever. Since that time every symptom of the complaint has entirely disappeared. His health is so far re-established, and daily acquires such an accession of strength, that he is no longer affected with heart-burn, indigestion, nervous attacks, or any symptoms of this description. He is capable of walking eight or ten miles, without resting himself, and without feeling any inconvenience from the exertion. It may be proper to observe, that that his corpulency is considerably reduced by the effect of these solvent remedies, as well as by exercise.

Another circumstance proves how much the constitution of the Abbé has been strengthened by a long exhibition of the remedies already described. During sixteen years, he had been subject to a chronic complaint, accompanied with almost every species of acrimony; but he had never been in the least affected with any bilious disorder. He has since, however, been obliged to have recourse to the juice of lemons, as a remedy for this complaint. The intense heat of the summer of 1782, and a diarrhoea, with which he was four times attacked in the months of July and August, and which he stopped by astringent and constipating diet, instead of employing aperient medicines, produced a bilious inflammatory fever. This fever relieved itself, in a great degree, by an erysipelas in the left leg; and the bile was no sooner evacuated, than the Abbé was obliged to resort to extract of hemlock and aconite, in order to correct the heart-burn occasioned by this indisposition.

The medicines produced their full effect, and his health is now more firmly re-established.

The Abbé Mann has thus faithfully recited an incontestable fact; the practical conclusions which may be inferred from it, he leaves to the judgment of others.

